# *Hhex* Is Necessary for the Hepatic Differentiation of Mouse ES Cells and Acts via *Vegf* Signaling

**DOI:** 10.1371/journal.pone.0146806

**Published:** 2016-01-19

**Authors:** Adam S. Arterbery, Clifford W. Bogue

**Affiliations:** Department of Pediatrics, Section of Critical Care Medicine, Yale School of Medicine, Yale University, New Haven, Connecticut 06520, United States of America; University of Kansas Medical Center, UNITED STATES

## Abstract

Elucidating the molecular mechanisms involved in the differentiation of stem cells to hepatic cells is critical for both understanding normal developmental processes as well as for optimizing the generation of functional hepatic cells for therapy. We performed *in vitro* differentiation of mouse embryonic stem cells (mESCs) with a null mutation in the homeobox gene *Hhex* and show that *Hhex*^*-/-*^ mESCs fail to differentiate from definitive endoderm (Sox17^+^/Foxa2^+^) to hepatic endoderm (Alb^+^/Dlk^+^). In addition, hepatic culture elicited a >7-fold increase in *Vegfa* mRNA expression in *Hhex*^*-/-*^ cells compared to *Hhex*^*+/+*^ cells. Furthermore, we identified VEGFR2^+^/ALB^+^/CD34^-^ in early *Hhex*^*+/+*^ hepatic cultures. These cells were absent in *Hhex*^*-/-*^ cultures. Finally, through manipulation of *Hhex* and *Vegfa* expression, gain and loss of expression experiments revealed that *Hhex* shares an inverse relationship with the activity of the *Vegf* signaling pathway in supporting hepatic differentiation. In summary, our results suggest that *Hhex* represses *Vegf* signaling during hepatic differentiation of mouse ESCs allowing for cell-type autonomous regulation of *Vegfr2* activity independent of endothelial cells.

**Highlights**

- *Hhex*^*-/-*^ ESCs fail to differentiate from definitive endoderm to hepatic endoderm
- This defect involves perturbation of VEGF signaling pathway
- Differentiation involving this pathway produces VEGFR2+ hepatic progenitor cells
- VEGF regulation of hepatic specification is independent of endothelial cells

## Introduction

The liver originates from the foregut definitive endoderm (DE), which forms from the mesendoderm of the anterior region of the primitive streak [1]. These endodermal precursors give rise to cells for both the liver and pancreas. DE movement is accompanied by epithelial-mesenchymal transition and the hepatic endoderm (HE) is specified and begins to bud from DE around embryonic day (E) 8.5–9.5 in the mouse [2]. Throughout development, liver growth is maintained by a population of progenitor cells called hepatoblasts [3]. These progenitor cells are thought to give rise to the two main cell types in the liver, hepatocytes and biliary cells. Interestingly, a growing body of evidence indicates that the adult liver has functional stem cells. These adult hepatic progenitor cells can differentiate, trans-differentiate, and trans-determine between multiple terminal cell fates of DE origin, including pancreas and intestine [4, 5]. More strikingly, the genetic mechanisms behind fetal and adult liver homeostasis are very similar [6]. Therefore, characterizing the genetic components of the liver’s ability for continued self-regeneration through multiple developmental stages is fundamental to understanding the biology of liver growth and regeneration. In addition, studies focused on progenitor cells rather than terminally-differentiated cells can offer unique insight into the genetic mechanisms underlying organogenesis [7]. In vitro ESC-derived HE cells offer great potential for the treatment of many liver diseases, can provide insight into processes involved in drug metabolism, and can provide important insight into congenital liver diseases. One of the main factors hindering progress in realizing the therapeutic potential of stem cell-derived liver progenitor cells is a core understanding of the molecular mechanisms involved in the early stages of hepatic commitment.

*Hhex*, also known as *Prh*, has been shown to have roles in many biological processes including cell cycle regulation, organ development, and cell differentiation via both transcriptional activation and repression [8]. During liver development, *Hhex* is first expressed broadly in the DE at E7.0 and then becomes restricted to the foregut endoderm one day later [9]. Around the time of liver budding (E8.5–9.0), *Hhex* expression in the foregut is primarily restricted to the ventral medial foregut, where the liver bud forms [10]. Currently, little is known about the genes and/or signaling pathways acting downstream of *Hhex* during hepatic specification and liver bud formation. However, *Hhex* has been shown to be involved in events prior to and just after specification. In *Hhex*^-/-^ mice, no liver forms and it has been reported that in these mice foregut development is normal and initial hepatic specification occurs, yet liver precursor cells fail to form a liver bud lined by a pseudostratified epithelium and to subsequently migrate into the adjacent septum transversum mesenchyme [7, 9, 11]. In another mouse model, targeted deletion of *Hhex* expression in the foregut and hepatic diverticulum at E8.5—E9.5 resulted in severe hepatic defects, including hypoplasia of the liver, absence of extra-hepatic and intrahepatic bile ducts, and evidence of an hepatoblast differentiation defect [12]. In addition, studies suggest that *Hhex* has transcriptional targets in ventral DE progenitor cells that influence their proliferation and that reduction of *Hhex* results in the loss of both liver and pancreatic gene expression [8, 13].

*Hhex* has been shown to repress the transcription of multiple Vegf signaling components including ligands and receptors during angiogenesis [14] and hemangioblast differentiation [15]. Furthermore, the absence of *Hhex* expression in the mouse embryo perturbs cardiovascular development due to an increase in Vegf levels [16]. The Vegf signaling pathway is most commonly associated with its well-known role in hematopoietic/endothelial cell differentiation. However, two previous studies have also suggested a potential link between Vegf signaling and hepatogenesis. Matsumoto et al. used a *Vegfr2*^*-/-*^ (also known as *Flk1* or *Kdr*) mouse to show that hepatic progenitor cells fail to migrate into the septum transversum in the absence of *Vegfr2* expression [17]. The authors concluded that the defect was due to a loss of endothelial cells during the early stages of liver organogenesis, leading to disrupted endodermal-endothelial communication and a failure of cell migration and liver bud formation. Additionally, a Vegfr2^+^ early hepatic progenitor cell was recently identified in both mice and humans that is capable of terminal differentiation into mature endodermal liver cell types (hepatocytes and biliary epithelial cells) [18]. The transcriptional mechanisms supporting Vegfr2-mediated hepatic progenitor differentiation were found to be cell autonomous.

How *Hhex* regulates hepatic differentiation, and if Vegf signaling is downstream of *Hhex* in this process, are both unknown. Thus, to address these gaps in our knowledge, we differentiated DE and HE progenitor cells from wild type and *Hhex*^*-/-*^ mouse ESCs and compared the molecular signatures that accompanied the transition of DE progenitor cells to cells of the hepatic lineage. We show that the absence of *Hhex* expression blocks HE differentiation, in part via a transcriptional pathway that involves Vegf signaling.

## Materials and Methods

### Materials

See S1–S4 Tables for tissue culture, antibodies, and qPCR materials.

### ESC Cultures

All animal work and sample collection in this study was done in accordance with protocols that were approved by the Yale University Institutional Animal Care and Use Committee. Cell culture was performed at 37°C with 5% CO_2_. *Hhex*^*-/-*^ Jet-BL6 ESCs were derived via homologous recombination, isolated, and obtained from the Yale Animal Genomics Service as previously reported [19]. DE and HE cells were derived as previously reported with some modification [20–22]. Briefly, ES cells were grown in ESC medium (See S1 Table for medium ingredients) on a MEF-feeder layer using a gelatin-coated 35mm petri dish, and grown to confluence. Cells were then re-plated and grown to confluence in adaptation medium on gelatin-coated plates. Embryoid bodies (EBs) were formed by plating 6x10^4 cells/mL from the confluent adaptation cultures into one well of an AggreWell 400 plate (Stemcell Technologies, Vancouver, CA) and further cultured in adaptation medium for 2 days. EBs were then harvested, replated on collagen coated culture dishes and allowed to expand for additional 2 days in adaptation medium (as described in [23] with some modification). After two days of expansion, the medium was switched to DE medium and EBs were cultured for another 5 days [24, 25]. Under these conditions, endodermal induction with 100 ng/mL of Activin A has been shown to yield ~30% DE cells [26] that are characterized by increased expression of Sox17, Gsc, and Foxa2/Hnf3b, as well as decreased pluripotency [27]. Following the 14 day DE differentiation protocol, cells were trypsinized, and: frozen down for low-passage preservation; subjected to RNA harvest; stained for FACS; subjected to immunohistochemical analysis (immunofluorescence staining); or replated on collagen coated culture dishes in HE media for 7–10 days to induce the differentiation of HE progenitor cells. Under these conditions, hepatic induction has been shown to yield 50–60% murine hepatic progenitor cells [18, 24]. At the end of the HE culture, cells were: frozen down for low-passage preservation; subjected to RNA harvest; stained for FACS; or subjected to immunohistochemical analysis (immunofluorescence staining). RNA samples were obtained using an RNeasy Kit and DNase treated with an RNase-Free DNase Kit according to the manufacturers protocol (both kits from Qiagen Inc. Valencia, CA). These samples were used for template in cDNA reactions and analyzed for gene expression using quantitative real-time PCR (qPCR). The qPCR and IF analyses were performed as indicated below.

### Quantitative Real Time PCR (qPCR)

cDNA was prepared from DNase treated RNA using QScript cDNa supermix (Quanta Biosciences, Gaithersburg, MD). Reactions were run in duplicate and analyzed using a 7900HT Fast Real Time PCR System (Applied Biosystems Foster City, CA) under the following cycling conditions: 50°C for 2 minutes; 95°C for 10 minutes; then 45 cycles of 95°C for 5 seconds followed by 30 seconds at 60°C (annealing temperature). Each QPCR reaction contained the following: 10 μL Perfecta SYBR GREEN Fast Mix with Rox (Quanta Biosciences, Gaithersburg, MD), 1 μl of forward and reverse primers, 2 μL cDNA, and 6 μL nuclease-free water. See S3 Table for a list of genes analyzed and primers used. qPCR was performed on both EB-derived cultures and sorted cells. Raw values were converted to absolute copy number using a standard curve covering a linear range of 5x10^6^ to 10 copies. Absolute copy numbers were then normalized to *Actb* and *Gapdh* and averaged. These normalized values were used for comparisons across all samples. We performed absolute quantification of mRNA expression using real-time PCR on the HE cultures with the gene expression data presented as *Hhex*^*+/+*^ HE cultures relative to *Hhex*^-/-^ HE cultures (normalized expression divided by normalized expression). See S3 Table for primer details. RNA was harvested from whole culture, not FACS isolated, cells for DE mRNA analysis.

For quantitative gene expression analysis of mouse embryonic livers, DNase treated RNA was isolated from E11.5 and E13.5 livers using Qiagen RNeasy kit (Qiagen, Valencia, CA) according to manufacturers protocol. The production of cDNA and the real-time PCR protocol were preformed as above.

### Immunofluorescence

Following optimization experiments, cells were washed in PBS, fixed in 4% paraformaldehyde for 15 minutes, permeabilized with PBS-0.05% Tween 20 (PBST) for 10 minutes, and blocked in PBST-1% BSA for 30 minutes. Primary antibodies (or IGG control antibodies) were diluted 1:100 in PBST-1% BSA and applied to coverslips overnight at 4C. The next day, the cells were washed in PBS and incubated with secondary antibody for 1 hour in the dark (Alexa Fluor 488 and 594—Invitrogen, Grand Island, NY). Following secondary incubation, the cells were washed in PBS, counterstained with Hoechst 33342 (Molecular Probes, Eugene, OR) at 0.5 μg/mL in the dark, and mounted to slides using Prolong Gold anti-fade reagent (Invitrogen, Grand Island, NY). Slides were dried overnight and visualized the next day on an Inverted upright Zeiss Axioscope, then stored at -4°C. Proteins used for antibody staining were as follows: DE staining—Foxa2 and Sox17; HE staining—Aat and Alb. See S2 Table for antibody information.

### Fluorescent Activated Cell Sorting (FACS)

Following optimization experiments, cells were trypsinized and blocked for one hour in PBS-1% BSA containing Fc Block (1ug/10^6 cells—BD Biosciences, San Jose, CA) on ice. Primary antibody [DE: Sox17^+^/Gsc^+^; HE: Alb^+^/Dlk^+^; hepatic progenitors: Vegfr2^+^/Alb^+^—see S2 Table for antibody information], or IGG control antibodies, were applied to cells for one hour at a concentration of 1:100, followed by the application of secondary antibody (Alexa Fluor 488, PEcy7, and Alexa Fluor 594—Invitrogen) for one hour at a concentration of 1:100. Cells were re-suspended in PBS containing pen/strep and gentamicin and kept at 4°C in the dark until sorted. Cells were sorted on a FACSAria (BD Biosciences, San Jose, CA) for a total of 1x10^6^ single cell events. To stain for intracellular markers, DE cultures were trypsinized, fixed in 4% paraformaldehyde for 15 minutes, permeabilized with PBS-0.05% Tween 20 (PBST) for 10 minutes, blocked for one hour in PBS-1% BSA containing Fc Block, stained, and FACS sorted.

### Treatments

At the end of DE and HE differentiation, respectively, cells were treated with recombinant protein or inhibitors for 72 hours and harvested for RNA as previously described. *Hhex*^*-/-*^ HE cells were re-plated, allowed to become confluent, and were then treated with either 30 ng/uL Vegf protein or vehicle. *Hhex*^*-/-*^ DE cells were re-plated in HE medium and grown to confluency and were then treated with either 10 uM Vegf inhibitor CBO-P11 or vehicle for control. To inhibit *Hhex* expression in *Hhex*^*+/+*^ HE cells, we used a combination of three predesigned siRNA oligos (IDTdna) in conjunction with the siRNA transfection reagent INTERFERin (Polyplus Transfection Inc.) according to the manufacturers instructions. Media was changed daily in all treatments. See S1 Table for protein and inhibitor details.

### Statistics

All statistical analysis was done using JMP software and Wilcoxon/Mann-Whitney analysis with Dunn analysis for joint ranks. Data were log transformed and subjected to Kruskal-Wallis post-hoc analysis when necessary.

## Results

### *Hhex* is not necessary for differentiation of mESCs to DE

**Fig 1:**
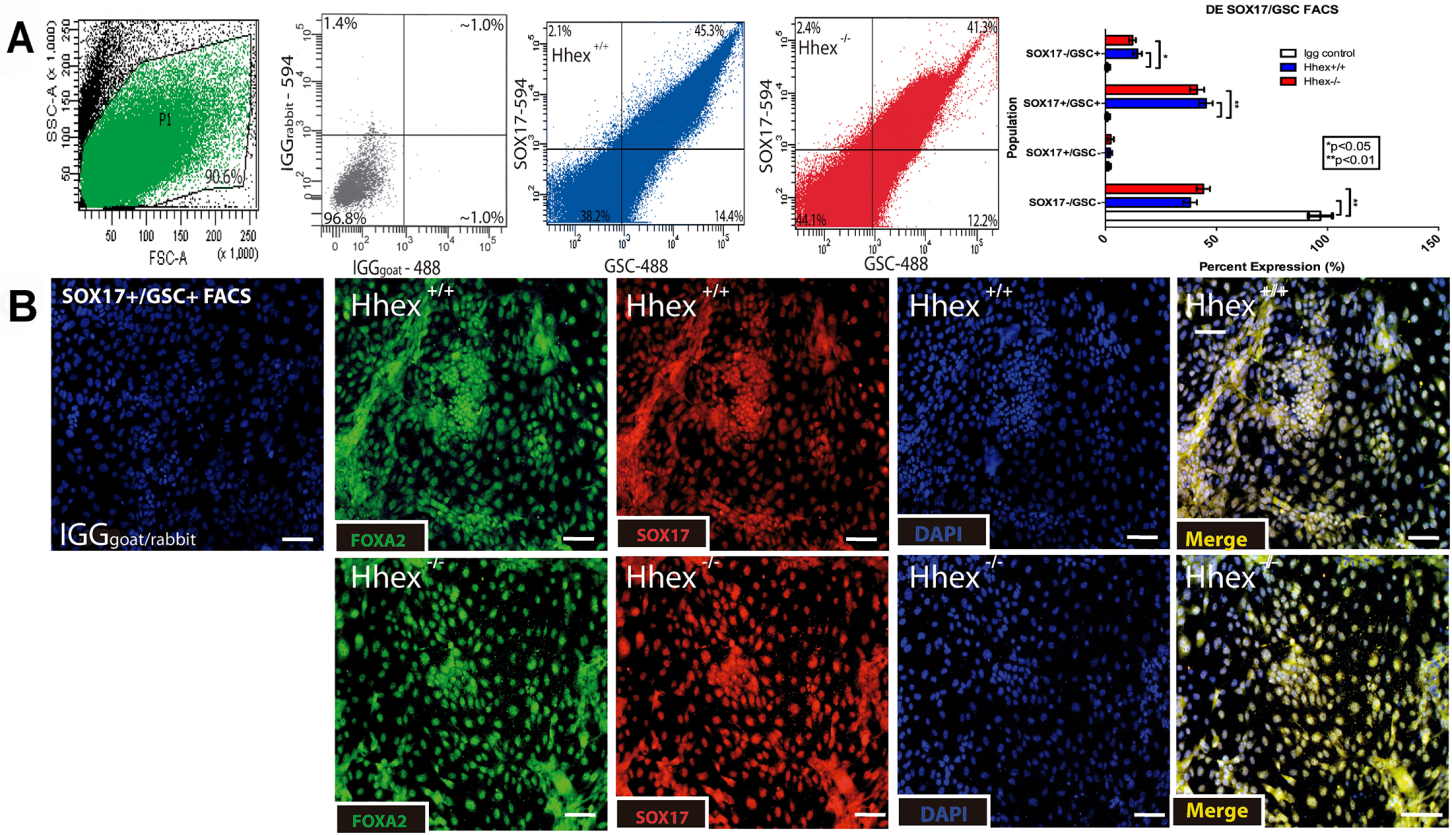
Differentiation of Definitive Endoderm. **Fig 1:** *Hhex*^*-/-*^ ESCs show no defects when differentiated toward definitive endoderm. **A)** Analysis of FACS for SOX17 and GSC revealed that both *Hhex*^*+/+*^ (blue) and *Hhex*^*-/-*^ (red) cultures produced similar percentages of differentiated DE cells. Light Scatter plot indicates the cells gated for sorting (green) and IGG plot (grey) confirms antigen specificity. **B)** Single channel and merged immunofluorescence staining of DE cultures after the 5-day culture. Both *Hhex*^*+/+*^ and *Hhex*^*-/-*^ cultures showed significantly more cells double positive for the nuclear expression of SOX17 and FOXA2/HNF3β compared to IGG controls. (Scale bars = 100μM, *p<0.05, **p<0.01)

**Fig 2:**
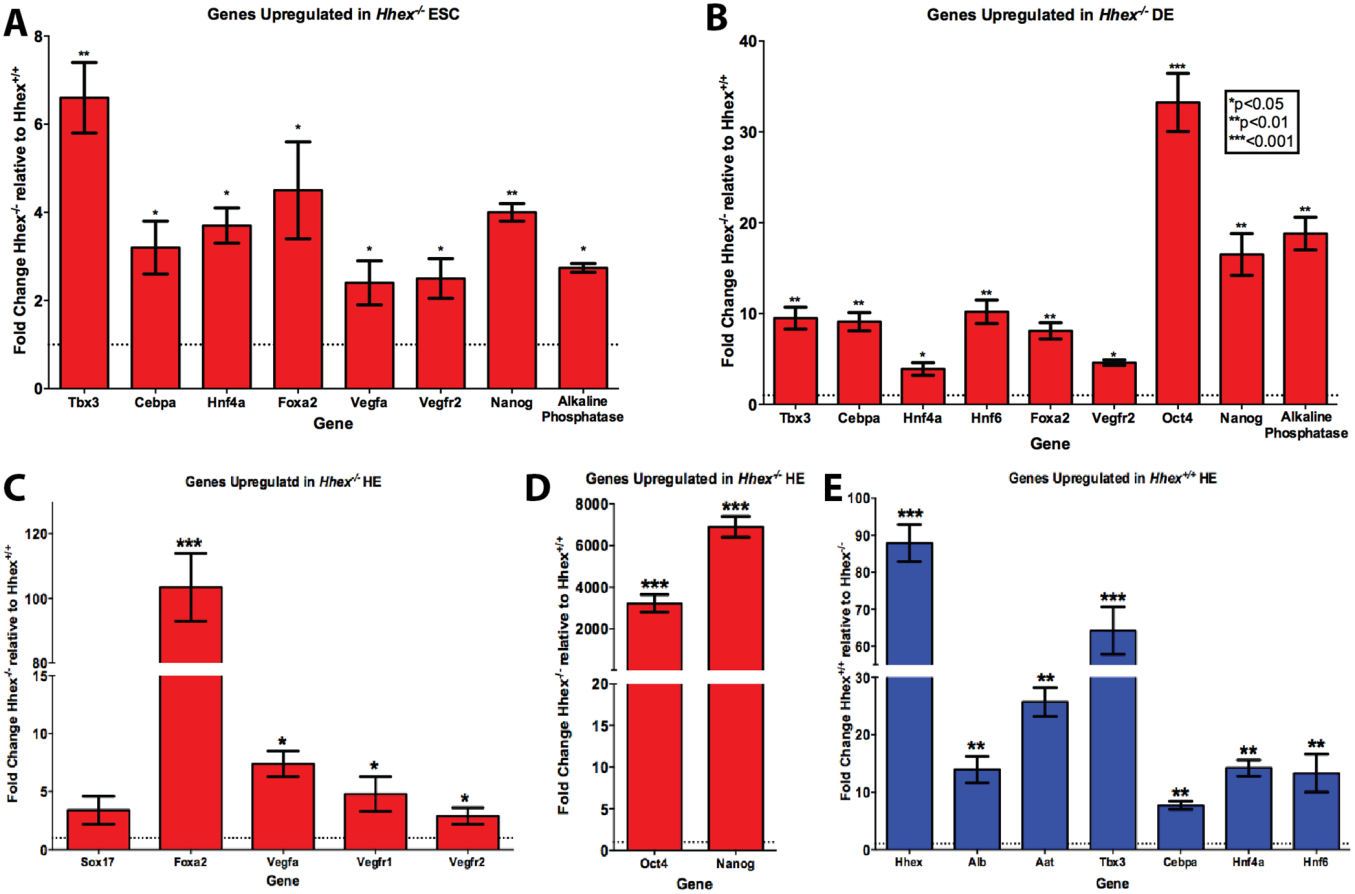
Genotype Comparison using QPCR at each Differentiation Stage. *Hhex*^*-/-*^ HE cells did not show mRNA expression consistent with hepatic differentiation. **A-E)** Comparison of fold-change in normalized mRNA gene expression using differentiation-stage specific markers. Comparison of pluripotency gene markers reveal *Hhex*^*-/-*^ (red) cells showed increased pluripotency relative to *Hhex*^*+/+*^ (blue) at each differentiation stage, particularly during HE differentiation **(A, B, and E)**. Comparison of definitive endodermal gene markers reveal *Hhex*^*-/-*^ cells fail to exhibit significant decreases in definitive endodermal gene expression characteristic of HE differentiation **(C)**, and as seen in *Hhex*^*+/+*^ HE cells. *Hhex*^*+/+*^ HE cells showed dramatic increase in hepatic gene expression **(D)**, while *Hhex*^*-/-*^ cells showed a heavily attenuated expression. Comparison of *Vegf* signaling gene markers showed that *Hhex*^*-/-*^ cells exhibit increased levels of ligands (*Vegf-a*) and receptor (*Vegfr1* and *Vegfr2*) gene expression at each differentiation stage, but particularly during HE differentiation **(A, B, and C)**. (Note raw data is presented in S1 Fig) (*p<0.05, **p<0.01, and ***p<0.001).

In the present report, we used cell culture methods employing embryoid body formation to differentiate ESCs to DE and DE to HE as previously reported, with some modification [20–22, 28]. Culture of cells produced from embryoid bodies in DE medium resulted in a highly differentiated population of DE cells that were SOX17+/FOXA2+ in both wild type and *Hhex*^*-/-*^ cultures (Fig 1 –protein expression; and Fig 2 –mRNA expression). SOX17 and FOXA2 are two proteins with high nuclear expression in definitive endodermal cells and are the most efficient markers for isolating definitive endoderrmal cell types [29, 30]. Thus, *Hhex* is not necessary for the differentiation of ESCs to DE. We also performed FACS for SOX17^+^/GSC^+^ cells on both *Hhex*^*+/+*^ and *Hhex*^-/-^ EB cell cultures at the end of the 5-day DE differentiation protocol. SOX17 and GSC are two proteins previously used to identify DE for cell sorting [26]. Similar to the use of the nuclear protein FOXP3 for lineage segregation of Tcells using FACS [31], the nuclear protein SOX17 has been used for lineage segregation of endoderm using FACS [32]. In *Hhex*^*+/+*^ DE cultures, 45.3% of the total population was SOX17^+^/GSC^+^ (Fig 1A) and in *Hhex*^-/-^ DE cultures, 41.3% of the total population was SOX17^+^/GSC^+^ (Fig 1A). Both of these percentages are consistent with previous reports on ESC-derived DE cells that used similar methods [33]. No dramatic differences were observed in the expression of mRNA genes between *Hhex*^*+/+*^ and *Hhex*^-/-^ DE cultures (Fig 2).

### *Hhex* is necessary for initiation of hepatic gene expression and repression of *Vegf* signaling in differentiation of DE to HE

**Fig 3:**
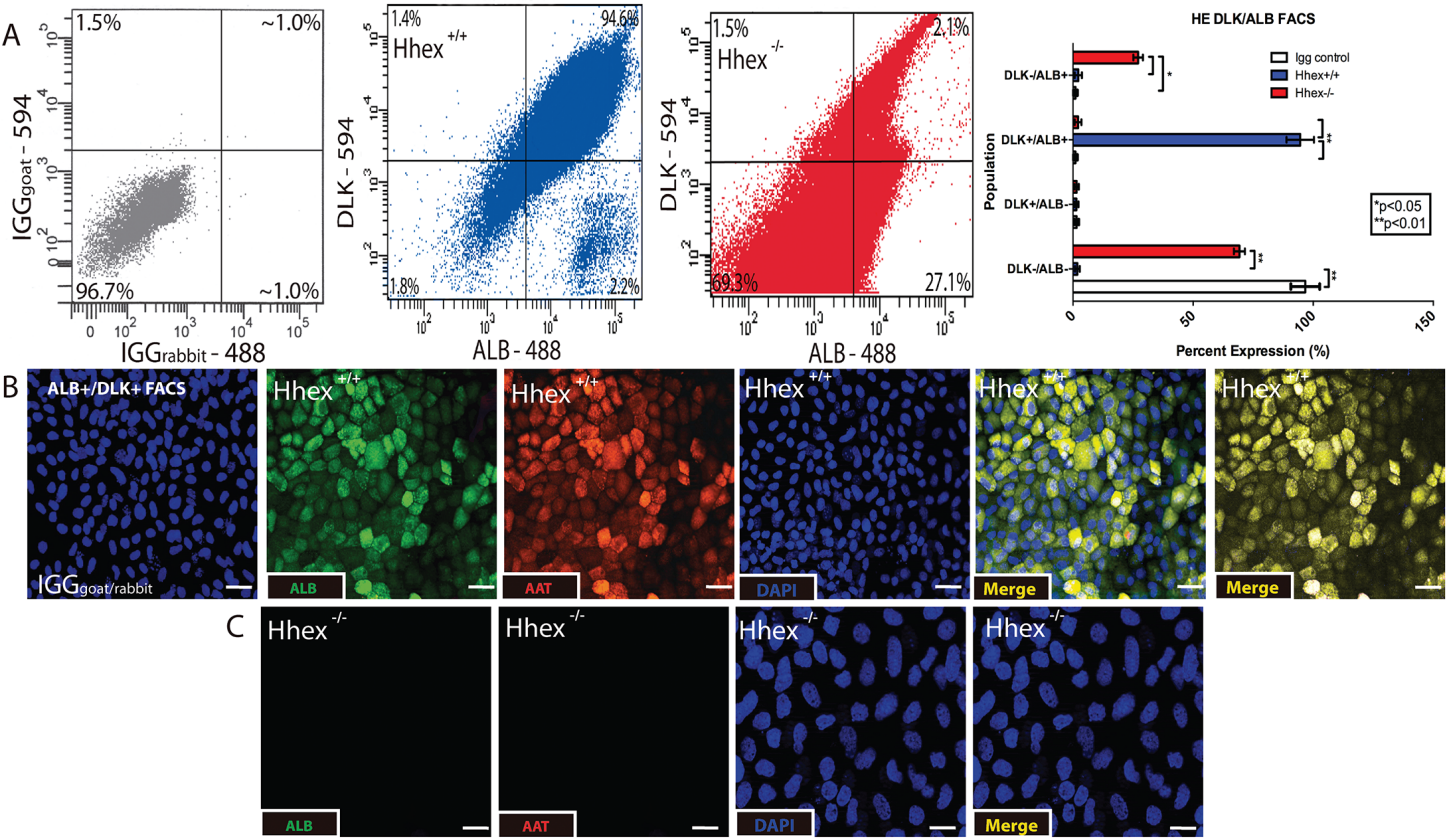
Differentiation of Hepatic Endoderm. *Hhex*^*-/-*^ definitive endodermal cells did not differentiate toward hepatic endoderm. **A)** Analysis of FACS for ALB and DLK revealed that only *Hhex*^*+/+*^ (blue) cultures produced significant populations of differentiated HE cells. IGG plot (grey) confirms antigen specificity. **B)** Single channel and merged immunofluorescence staining of HE cultures. Only *Hhex*^*+/+*^ cells showed a uniform and highly differentiated population of HE cells that were double positive for ALB and AAT compared to IGG controls. (Scale bars = 50μM, *p<0.05, **p<0.01).

Previous studies have investigated the differentiation of mouse ESCs toward HE using various methods [20–22]. *Hhex* has been implicated in the process as evidenced by low expression levels of *Alb* and *Afp* in *Hhex*^*-/-*^ ESCs differentiated towards the hepatic lineage [28]. After differentiation of DE cells from both *Hhex*^*+/+*^ and *Hhex*^-/-^ ESCs, we re-plated the DE cells in HE media and cultured for an additional 7–10 days to obtain differentiated hepatic progenitor cells. After 7–10 days in HE medium, cells were subjected to FACS using ALB and DLK, a combination of proteins previously used to identify and sort HE progenitor cells [34]. Culture of *Hhex*^*+/+*^ DE cells in HE medium for 10 days produced a population of differentiated cells that were 96.4% double positive for both ALB and DLK (Fig 3A). However, culture of *Hhex*^-/-^ DE cells in HE medium for 10 days did not result in the differentiation of a large cell population that was double positive for ALB and DLK (Fig 3A). In addition, immunofluorescence analysis revealed that the 10 day *Hhex*^*+/+*^ cultures exhibited a highly differentiated population of cells positive for both ALB and AAT, two proteins commonly used to identify hepatic progenitor cells [35] (Fig 3B). As expected, *Hhex*^-/-^ ESCs did not produce any observable population of ALB^+^/AAT^+^ cells (Fig 3C). Furthermore, *Hhex*^*+/+*^ HE cultures showed significantly higher mRNA levels of genes whose expression is known to increase during HE differentiation, including: 87.8-fold higher expression of *Hhex* (in *Hhex*^*+/+*^ cells when HE cultures are compared to DE cultures), 13.9-fold higher expression of *Alb*, 25.7-fold higher expression of *Aat*, 7.7-fold higher expression of *Cebpα*, 14.2-fold higher expression of *Hnf4α*, and 64.2-fold higher expression of *Tbx3*, and 13.3-fold higher expression of *Hnf6* (Fig 2E for genotype comparison within each differentiation stage, and S1 Fig for differentiation stage comparison within genotype). We also analyzed mRNA quantity for *Vegfa* and observed that *Hhex*^-/-^ sorted HE cells maintained significantly higher levels of expression for *Vegfa* (7.4-fold) and its receptors *Vegfr2* (2.9-fold) and *Vegfr1* (4.8-fold) (Fig 2C for genotype comparison within each differentiation stage, and S1 Fig for differentiation stage comparison within genotype). Finally, in addition to being absent for ALB and AAT (S1 Fig), *Hhex*^*-/-*^ HE cultures exhibited increased expression of DE genes and increased expression of pluripotency genes (Fig 2C and 2D for genotype comparison within each differentiation stage, and S1 Fig for differentiation stage comparison within genotype). Thus, upon differentiation of *Hhex*^-/-^ mESCs from DE to HE, the expression of Vegfa is increased, while the expression of liver-enriched genes is markedly attenuated compared to wild type cells. These results provide evidence for concurrent regulation of *Hhex*, HE genes, and *Vegfa* during the initial stages of HE differentiation in vitro.

In summary, comparison of mRNA expression between DE and HE stages for *Hhex*^*+/+*^ and *Hhex*^*-/-*^ cells indicated that the failure of HE differentiation from DE progenitor cells in *Hhex*^*-/-*^ ESCs. This was characterized by a failure to increase HE gene expression (*Hhex*, *Alb*, *Aat*, *Cebpα*, *Hnf4α*, *Tbx3*, and *Hnf6*), maintenance of high levels of DE gene expression, and dramatically increased expression of the pluripotency marker gene *Oct4*, *Nanog*, and *Alkaline phosphatase*). It should be noted that Alkaline phospahatse was chosen as a marker for pluripotency, as a high expression of SOX2 has been reported in cells driven toward the endodermal lineage using Activin A (SOX17+ cells) [36]. Additionally, this is accompanied by a significant increase in *Vegfa*, *Vegfr1*, and *Vegfr2* expression.

### ESC-derived hepatic precursor cells express VEGFR2

**Fig 4:**
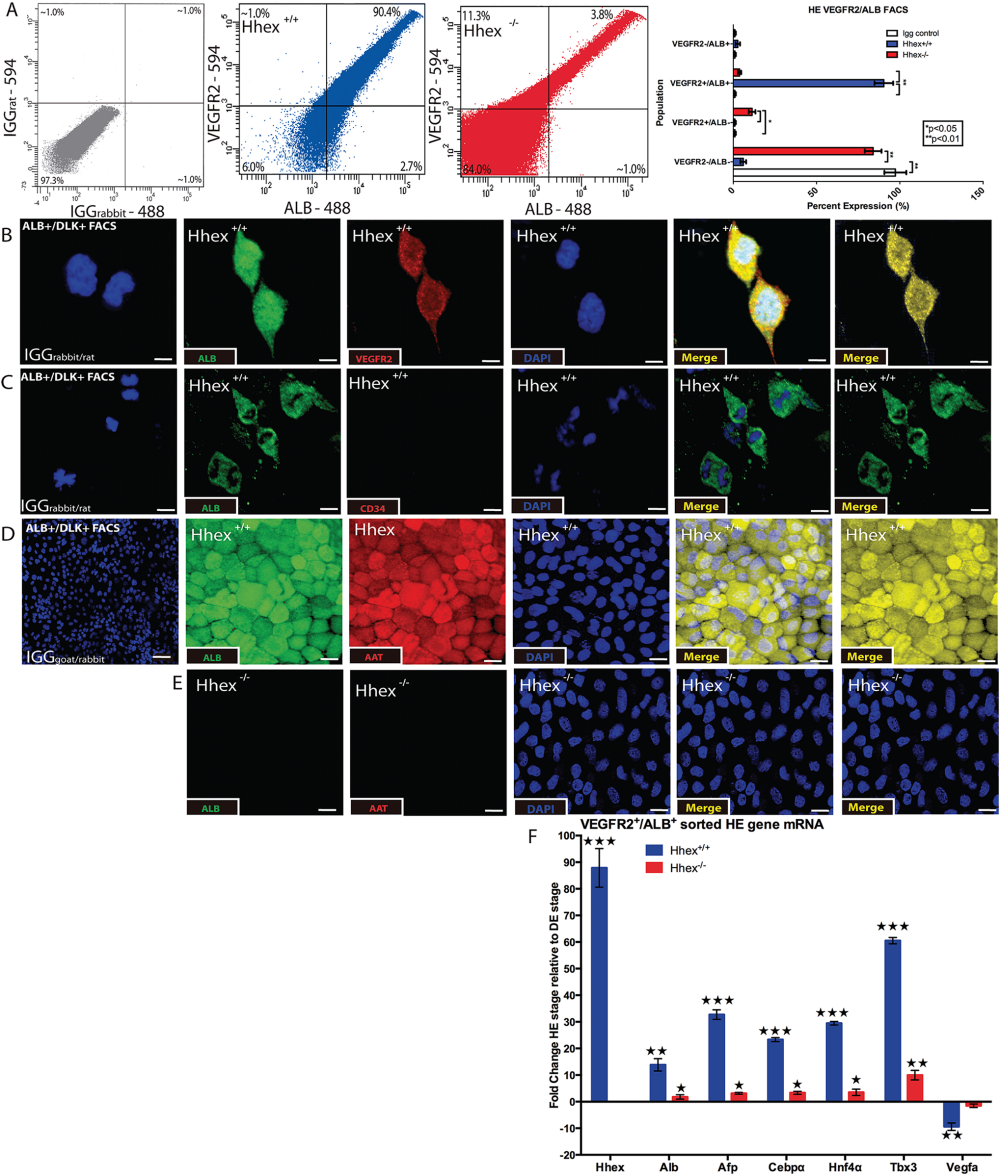
Differentiation of VEGFR2+ Hepatic Endoderm. *Hhex^-/-^* DE cells did not differentiate into VEGFR2^+^ early hepatic progenitor cells. **A)** Analysis of FACS for ALB and VEGFR2 revealed that only *Hhex*^*+/+*^ (blue) cultures produced significant populations of HE progenitor cells and the majority of ALB^+^ cells were also VEGFR2^+^ in *Hhex*^*+/+*^ cultures. IGG plot confirms antigen specificity. **B and C)** Single channel and merged immunofluorescence staining of *Hhex*^*+/+*^ ALB^+^/VEGFR2^+^ sorted cells that were replated for 24 hours in HE media. Sorted cells showed co-expression of ALB and VEGFR2 (B), and were absent for the expression of the hematopoietic/endothelial marker CD34 (C). (Scale bars = 10μM.) **D and E)** Single channel and merged immunofluorescence staining of *Hhex*^*+/+*^ and *Hhex*^*-/-*^ ALB^+^/VEGFR2^+^ sorted cells that were replated for 7 days in HE media. Despite rapid expansion of the *Hhex*^*-/-*^ sorted cells, only *Hhex*^*+/+*^ sorted cells showed a co-expression of ALB and AAT that is indicative of further/continued hepatic differentiation. (Scale bars = 50μM.) **F)** Normalized mRNA expression of hepatic and *Vegf* signaling gene markers in ALB^+^/VEGFR2^+^ sorted cells from both *Hhex*^*+/+*^ and *Hhex*^*-/-*^ cultures. *Hhex*^*-/-*^ sorted cells show heavily attenuated mRNA expression for hepatic genes and no significant reduction in *Vegfa* expression when compared to cells from the previous DE differentiation stage. (*p<0.05, **p<0.01, ***p<0.001).

Our data suggest that *Vegfa* mRNA levels are inversely correlated with HE differentiation and are increased in the absence of *Hhex* (see results above). Based on these data, we looked for the presence of VEGFR2^+^ murine hepatic progenitor cells in *Hhex*^*+/+*^ and *Hhex*^*-/-*^ HE cell cultures. As recently reported, VEGFR2^+^ hepatic progenitors are amongst the earliest cells to differentiate from the DE stage toward the HE stage [18]. In addition, Vegfr2 is known to stimulate the majority of transcriptional activity in response to changes in Vegfa expression [37]. After one day of HE culture, *Hhex*^*+/+*^ cells exhibited expression of VEGFR2 and ALB protein as assessed by immunofluorescnece (Fig 4B). This indicates that HE cells express VEGFR2 very early during in vitro differentiation, and are thus capable of utilizing Vegf signaling. CD34 is a marker for hematopoietic/endothelial cells, and was not observed to be co-expressed on ALB^+^ cells (Fig 4C). These results suggest that *Hhex*^*+/+*^ HE cells are not likely of endothelial origin and that there is no contamination of endothelial cells in our differentiated populations. While we do not see an indication of differentiation of *Hhex*^-/-^ cells toward endothelial lineage under HE culture conditions, it is possible that *Hhex*^-/-^ cells might more easily form endothelial cells under proper culture conditions. However, due to the reported role of *Hhex* in endothelial differentiation [38], endothelial maturation might be defective as well. We did not observe a significant amount of endothelial activity as a result of VEGF expression under HE conditions, however, the media conditions may preclude the differentiation toward the endothelial lineage despite a potential predisposition to do so. At the end of HE culture, these same VEGFR2^+^ *Hhex*^*+/+*^ hepatic cultures exhibited robust expression of ALB and AAT protein as assessed by immunofluorescence (Fig 4D). In addition, *Hhex*^-/-^ HE cultures did not show any observable ALB or AAT expression (Fig 4E). Upon FACS analysis of *Hhex*^*+/+*^ HE cells for both VEGFR2 and ALB, we observed many double-positive cells. In fact, 90.4% of *Hhex*^*+/+*^ HE cells were ALB^+^/VEGFR2^+^ while ALB^+^/VEGFR2^-^ cells represented only 3.8% of the total population (Fig 4A). We then compared gene expression of both *Hhex*^*-/-*^ and *Hhex*^*+/+*^ ALB^+^/ VEGFR2^+^ sorted cells to their respective DE cell population and found, as expected, that *Hhex*^*-/-*^ cells had very attenuated increases in hepatic gene expression with no change in *Vegfa* expression (Fig 4F). On the other hand, *Hhex*^*+/+*^ cells had large increases in hepatic gene expression accompanied by a large decrease in *Vegfa* expression. In summary, the vast majority of *Hhex*^*+/+*^ ESC-derived hepatic precursor cells express VEGFR2. The absence of *Hhex* impairs the differentiation of DE cells to HE and this is accompanied by dramatic perturbations in *Vegfa* and Vegfr2 expression.

### *Hhex* expression is critical for the regulation of *Vegf* in HE differentiation and maintenance of HE gene expression

**Fig 5:**
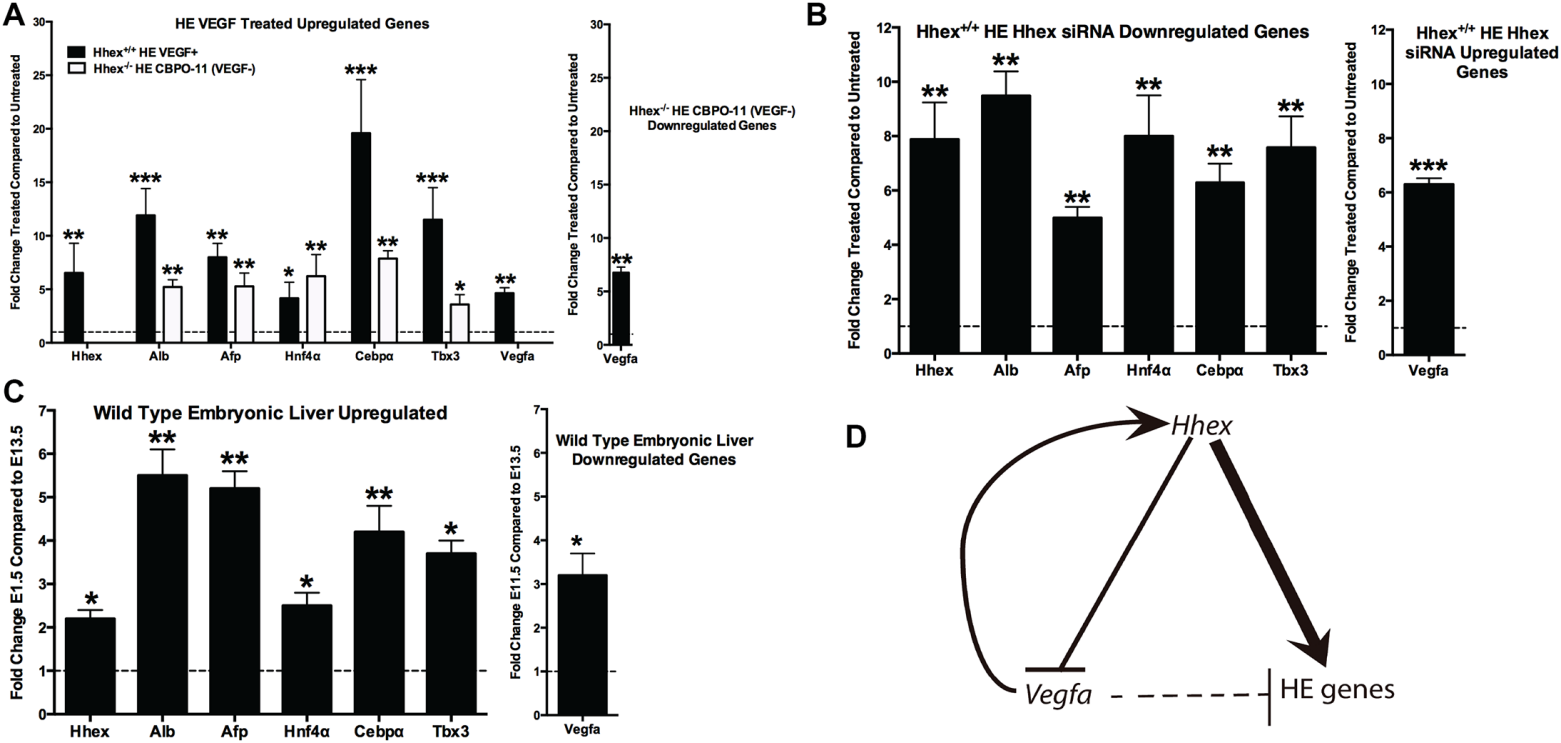
Proposed *Hhex-Vegf* Hepatic Regulatory Pathway. *Hhex* and *Vegf* signaling modulate the commitment of DE cells toward the HE lineage. **A)** The addition of VEGFA protein to *Hhex*^*+/+*^ HE cells facilitated HE differentiation as indicated by increased hepatic gene marker expression (black bars). Similar results were seen with the reduction of VEGF signaling in *Hhex*^*-/-*^ DE cells cultured in HE media (white bars). **B)** The reduction of *Hhex* using siRNA in *Hhex*^*+/+*^ HE cells resulted in decreased expression of hepatic gene markers and increased *Vegfa* expression. **C)** Comparative qPCR analysis for the expression of hepatic markers during the stages of mouse embryonic hepatic expansion, E11.5-E13.5. Hepatic markers change similarly *in vivo* when compared to the results obtained from the *in vivo* differentiation from ESC in the current report. **D)** We propose an *Hhex*-regulated model of HE specification whereby *Hhex* is necessary for the expression of hepatic genes, in part, via reducing/regulating *Vegf* signaling. (* = p<0.05, **p<0.01, ***p<0.001).

To determine the role of *Hhex* in regulating Vegf signaling during HE differentiation, we treated day-one HE cultures with 1) CBO-P11 to decrease Vegf signaling in *Hhex*^*-/-*^ cells, and 2) exogenous VEGFA to increase Vegf signaling in *Hhex*^*+/+*^ cells (Fig 5A). CBO-P11 is a 17-amino acid peptide derived from the region of the Vegf peptide that mediates binding to its receptors and blocks the binding of VEGFA to its cognate receptors VEGFR1 and VEGFR2 [39]. Inhibition and activation of the Vegf signaling pathway, such as with CBO-P11, has been previously shown to modulate the induction of VEGF secretion [40, 41]. Accordingly, inhibition of Vegf signaling with CBO-P11 is expected to decrease VEGFA/Vegfa secretion/expression in the current model and allow for the examination of the effect of reduced cellular stimulation from VEGF. When we inhibited Vegf signaling in *Hhex*^-/-^ HE cells using CBO-P11 (10uM for 72 hours resulting in a significant down-regulation of *Vegfa* 6.75-fold), we observed significant increases in HE gene expression: 5.21-fold increase in *Alb*, 5.27-fold increase in *Aat*, 6.22-fold increase in *Cebpα*, 7.87-fold increase in *Hnf4α*, and 3.58-fold increase in *Tbx3* expression (Fig 5A). Unexpectedly, when VEGFA was added to *Hhex*^*+/+*^ HE cells at 30 ng/uL for 72 hours (resulting in a significant up-regulation of *Vegfa* expression 4.84-fold), *Hhex* expression increased 6.52-fold and was accompanied by significant increases in the expression of other HE genes: 11.91-fold increase in *Alb*, 7.99-fold increase in *Aat*, 4.16-fold increase in *Cebpα*, 19.59-fold increase in *Hnf4α*, and an 11.50-fold increase in *Tbx3* expression (Fig 5A). Thus, in *Hhex*^*-/-*^ cells, a decrease in HE gene expression is accompanied by elevated *Vegfa* levels due to the loss of *Vegfa* repression by *Hhex*. When Vegf signaling is blocked in *Hhex*^*-/-*^ cells by CBO-P11 treatment, HE gene expression is rescued, demonstrating an important inhibitory role for Vegf signaling in HE gene expression. Interestingly, the expected decrease in HE gene expression upon treatment with exogenous VEGFA is blocked when *Hhex* is present, indicating that the positive effect of *Hhex* on HE gene expression can overcome any inhibitory influence on this process by *Vegfa*.

To explore if *Hhex* regulates hepatic gene expression and Vegf signaling in ESCs that have already been differentiated to HE, we treated 10 day-old *Hhex*^*+/+*^ cultures with a combination of three *Hhex* siRNA oligos to knockdown *Hhex* expression (Fig 5B). Upon treatment of *Hhex*^*+/+*^ HE cells with a combination of three siRNA oligos targeted to *Hhex* at 1uM each for 72 hours, we observed a significant decrease in the expression of HE genes: 7.87-fold decrease in *Hhex*, 9.48-fold decrease in *Alb*, 4.99-fold decrease in *Aat*, 7.99-fold decrease in *Cebpα*, 6.28-fold decrease in *Hnf4α*, and a 7.57-fold decrease in *Tbx3* (Fig 5B). In addition, as expected, we saw a 6.29-fold increase in *Vegfa* expression (Fig 5B). These data are consistent with the gene expression patterns we observed in *Hhex*^-/-^ HE cells and support a *Hhex*-dependent mechanism in the post-specification maintenance of HE gene expression via Vegf signaling. These results also support the regulation of hepatic differentiation by both *Hhex* and *Vegfa* and suggest that *Vegfa* provides feedback input to increase *Hhex* expression (suggesting the presence of an inverse regulatory relationship—see Fig 5D). Treating *Hhex*^*+/+*^ HE cells with exogenous VEGFA resulted in increased HE gene expression that was accompanied by an increase in *Hhex* expression. In summary, we found that in the presence of *Hhex*, *Vegfa* acts as a stimulator of HE gene expression by increasing *Hhex* expression. However, in the absence of *Hhex*, *Vegfa* acts as a repressor of HE gene expression. Thus, while both *Hhex* and *Vegfa* effect HE gene expression, the positive effect of *Hhex* on hepatic differentiation is dominant over the inhibitory effect of *Vegfa* (Fig 5D).

Finally, to confirm that the gene expression pattern observed in the current *in vitro* report is relevant to *in vivo* mechanisms, we subjected liver from embryonic mice (embryonic days E11.5 and E13.5) to qPCR assessment for hepatic markers. In the mouse, the liver is mainly a hematopoietic organ prior to embryonic day E12.5 [42], thus comparison of gene expression prior to and shortly after E12.5 will reflect the early stages of hepatic (metabolic) function of the organ. As expected, the results for the in vivo analysis reflect gene expression events occurring in the in vitro model here (namely increased hepatic gene expression–*Hhex*, *Alb*, *Aat*, *Hnf4a*, *Cebpa*, and *Tbx3*, and decreased *Vegf* expression) (Fig 5C). These results parallel the gene expression pattern seen in *Hhex*^*+/+*^ HE cultures (Figs 2 and 5, and S1 Fig), but is absent in *Hhex*^*-/-*^ HE cultures.

## Discussion

The current results indicate that *Hhex* represses *Vegf* signaling during the differentiation of HE progenitor cells and that in the absence of *Hhex*, ESC-derived DE cells do not differentiate toward HE cells. Furthermore, we show that *Hhex* is necessary for the initial increase in hepatic gene expression during early HE differentiation in vitro and this is accompanied by a decrease in *Vegfa* expression, suggesting that *Vegfa* expression must decrease in order for hepatic differentiation to proceed. We confirmed this finding by showing that inhibition of Vegf signaling in *Hhex*^*-/-*^ cells, in which *Vegfa* levels are markedly increased, rescued the molecular signature of HE differentiation. When we treated *Hhex*^*+/+*^ HE cells with VEGFA to increase Vegf signaling, we saw an increase in HE gene expression accompanied by an increase *Hhex* expression. This indicates that the inhibitory effect of *Vegfa* on HE differentiation is blocked in the presence of elevated *Hhex* levels. Taken together, these results indicate that the repression of Vegf signaling via *Hhex*, which occurs in hematopoietic cells [14, 38, 43, 44], supports the commitment of DE cells toward the HE lineage. However, Vegf-mediated regulation of HE gene expression may rely on both *Hhex*-dependent and *Hhex*-independent mechanisms.

*Hhex* has previously been linked to hepatic development both in vivo [7, 9, 11, 45] and in vitro [28]. *Hhex*^*-/-*^ embryos fail to develop a liver bud [11, 45] and it has been suggested that DE cells specify toward HE but fail to migrate and proliferate [7, 9]. However, these conclusions are based on the expression of genes not essential, nor absolutely specific for hepatic differentiation (such as *Alb*, *Afp*, *Foxa2*, and *Prox1*). For instance, it has been previously suggested that neither the expression of *Foxa2* [46] nor *Prox1* is specific for hepatic differentiation [47]. Also, previous studies have shown that *Afp* is expressed in other foregut derivatives such as gastrointestinal and pancreatic tissues [48, 49]. In addition, using *Alb* expression alone to define hepatic specification events has recently been questioned [50] and it has been shown that gut cells are capable of producing *Alb* transcripts in response to stimulation by *Gata4* [51]. However, when these genes are used in combination with other genes whose expression is more specific for the hepatic lineage (such as *Aat*, *Hnf4α*, *Cebpα*, and *Tbx3* as used in this study), a more accurate assessment of hepatic differentiation events.

It has been suggested that *Hhex* expression is correlated with hepatic specification events both in vivo [52–54] and in vitro [28]. Furthemore, it has been reported that hepatic cells from *Hhex*^*-/-*^ embryos show morphological defects related to liver organogenesis [7, 9]. In support of conserved function for *Hhex* regulation of HE specification, the use of *Hhex* morpholinos in zebra fish results in similar hepatic defects as those seen in mouse [55]. Overexpression of *Hhex* in DE cells has been suggested to induce HE gene expression while prolonged *Hhex* expression beyond HE specification has been suggested to be shift cell fate towards other cell types derived from and deter commitment toward the HE lineage [28]. In contrast to previous studies that used genetic manipulation instead of chemically defined conditions to induce hepatic differentiation, we studied ESC differentiation to HE by employing a commonly-used HE differentiation protocol [20–22]. Our results suggests that a loss of *Hhex* expression (not prolonged expression) just after HE specification, via treatment with siRNA, results in deterioration of the commitment toward a hepatic cell fate, as evidenced by a large decrease in the expression HE genes. Taken together, these results illustrate the complex nature of *Hhex* regulation of HE specification and differentiation. Concerning *Hhex* and its potential relationship to pluripotency, it has been well documented that increases in lineage determinant genes (such as *Hhex*) are paralleled by decreases in pluripotency genes (such as *Oct4*, *Nanog*, and *Alkaline Phosphatase* which are required for germ line transmission in mouse ESCs) [56]. However, it still remains to be determined what the mechanistic relationship between lineage determinants and pluripotency markers on the same cell. A growing body of evidence suggest that increased expression of pluripotency genes after lineage commitment may be representative of the labile nature for multi-lineage potential of pluripotent cells [57]. It should be noted that we observed increased expression of pluripotency markers at all stages in the *Hhex*^*-/-*^ mESCs when compared to *Hhex*^*+/+*^ mESCs from the same stage, although most notably in the HE stage (Fig 2). Thus, while our results suggest the *Hhex*^*-/-*^ mESCs acquire typical DE gene expression as seen in *Hhex*^*+/+*^ mESCs, pluripotency genes remain elevated representing a possible disconnect between DE gene expression, differentiation, and/or pluripotency genes. However, it has been previously reported that treatment with *Hhex* shRNA does not impair the progressive down-regulation of Sox2 and Oct4 during the induction of primitive and precardiac mesoderm [58]. In addition, we now report that *Hhex* has an important role in the induction of Vegf-regulated mechanisms that facilitate the specification, differentiation, and continued hepatic commitment of HE cells from DE progenitors.

*Vegfa* is the main ligand for stimulating the *Vegf* signaling pathway and mRNA levels for multiple *Vegf* signaling components (*Vegfa*, *Vegfr1*, *Vegfr2*) were higher in *Hhex*^-/-^ HE cells than in *Hhex*^*+/+*^ HE cells. Our results support a reciprocal relationship between *Hhex* and *Vegfa* in HE cells in which increased *Hhex* expression represses Vegfa and increased Vegfa increases *Hhex* expression [8, 14]. A similar relationship is known to exist in endothelial cells [14, 15], and therefore may represent a conserved *Hhex*-*Vegfa* molecular pathway that is shared between hepatic and endothelial cells. Activation of *Vegf* signaling can occur as the result of stimulation from multiple transcriptional regulators, including *Hhex*, which has been shown to act as both a direct [14, 43] and indirect [59] repressor of *Vegf* signaling activity. *Hhex* can induce transcriptional activation or repression via binding to a core consensus binding site motif (5’-C/tA/t**ATTA**AA/g-3’) [8, 60, 61]. Furthermore, *Hhex* has also been shown to bind non-consensus sites within target genes including *Vegf*, *Vegfr1*, and *Vegfr2* [14, 43]. In addition to these direct transcriptional repression mechanisms, *Hhex* has also been implicated via indirect repression where it has been shown to prevent transcriptional activation of the *Vegfr2* gene by inhibiting the binding of *Gata2* to its promoter regions [59].

As discussed earlier, most of the evidence for transcriptional interaction of *Hhex* with *Vegf* signaling comes from the hematopoietic literature (hematopoietic differentiation and leukemia). The predominate effect of *Hhex* in hematopoietic and leukemic cells is to regulate cellular growth [14, 38, 44], as high levels of *Hhex* expression lead to cell death and decreased *Vegf* signaling [14, 62]. Similarly, decreasing *Hhex* expression promotes cell growth via interruption of *Vegf* signaling-dependent apoptosis [14]. Reduction or absence of *Hhex* expression may sensitize cells to changes in *Vegf* signaling since *Hhex* is not present to modulate *Vegf* signaling. Therefore, the growth of cells with decreased or absent *Hhex* expression is likely to be shifted from an *Hhex*-dominant mechanism to a *Vegf*-dominant mechanism. Under this scenario, HE gene expression could be facilitated by both an increase in *Hhex* expression and a reduction in *Vegf* expression, each of which is mechanistically related to the other and results in combined effects on HE lineage commitment. In fact, loss of *Hhex* expression within myeloid cells results in the appearance of AML subtypes and CML blast crisis, both of which are hallmarks of abnormalities in the *Vegf*-signaling pathway [43, 63, 64]. The molecular interactions between *Hhex* and *Vegf* may also apply to cell types outside of the hematopoietic compartment, including thyroid and mammary epithelial cells [14, 65, 66].

While a role for *Hhex* in hepatic differentiation has been established by a number of investigators, it has been unclear, until recently, what role the *Vegf* pathway plays in hepatocyte specification/maturation, if *Hhex* and *Vegf* interact in the process, and if this interaction occurs between endothelial and endodermal cells or in endodermal cells alone. In 2009, Matsumoto et al. showed that the liver bud failed to form in *Vegfr2*^-/-^ embryos due either to the lack of signaling from, or absence of, endothelial cells [17]. They showed that at the time of *Hhex* expression and function, *Vegfr2* is required for the outgrowth and expansion of hepatic endodermal explants. However, this defect could be the absence of *Vegfr2* expression on endodermal cells as well as endothelial cells. Importantly, the analysis from that study showed that in early hepatic endoderm tissue explants, VEGFR2 and ALB expression overlaps. Thus, *Vegf* signaling may be intrinsic to both endothelial and hepatic cell types.

Interestingly, a recent report identified an in vivo population of non-endothelial, Vegfr2-positive cells (VegfR2^+^/CD31^-^), that were isolated from murine liver using lineage tracing [18]. These cells were shown to be hepatic progenitor cells that support the commitment/maturation of other hepatic progenitors (e.g.,Vegfr2^-^/CD31^-^) by increasing the expression of HE genes. This maturational effect seems to occur cell autonomously within the VEGFR2^+^ murine hepatic progenitor population. An endothelial cell population (VegfR2^+^/CD31^+^) was also isolated but did not support hepatic commitment/maturation. These VegfR2^+^/CD31^-^ hepatic progenitors are true progenitors from which functional hepatic cells (both hepatocytes and biliary epithelial cells), from both in vivo and in vitro sources, can be derived. The authors note that these cells marked for HE specification may differentiate into functional and terminal liver cell types via a progressive down-regulation of VEGFR2 (potentially via an *Hhex* mechanism). This recent report clearly establishes that *Vegfr2*, which has traditionally been defined as a mesodermal and hematopoietic/endothelial/vascular marker, is also expressed on, and plays a role in, HE progenitor cells that function to instruct early liver development via both non cell-autonomous (paracrine) and cell-autonomous (autocrine) signaling.

Our results are in agreement with the study by Goldman et al (identification of VEGFR2^+^/ALB^+^/CD34^-^ murine hepatic progenitor cells) and provides strong support for the notion that *Vegf* signaling is intrinsic to the HE cell and independent of the presence of endothelial cells [18]. Additionally, *Vegf* signaling in foregut epithelial cells has previously been proposed to be independent of an endothelial cell function [67–69]. While Vegf/Vegfr2 expression/activation has traditionally restricted to mesodermal and ectodermal derivatives, we now offer further support Vegf signaling as a marker for endodermal derivatives as well. Therefore we propose that *Hhex* facilitates HE specification and differentiation (continued commitment) from DE progenitors in part via an intrinsic *Vegfa*-*Vegfr2* mechanism.

## Conclusions

In summary, our investigations suggest a novel transcriptional relationship between *Hhex* and *Vegf* signaling in HE differentiation. We propose that *Hhex* is required for the onset of HE differentiation and that *Hhex* stimulates the expression of many hepatic genes. Its absence perturbs the hepatic competence of differentiating DE cells in part by failing to reduce *Vegf* signaling activity. In addition, we observed that *Vegfa* affects HE differentiation independent of *Hhe*x. From the current results, and those previously reported, *Hhex* is very likely to be upstream of *Vegf* signaling in hepatic cells. Furthermore, the failure to attenuate hepatic gene expression following *Hhex* inhibition in *Hhex*^*+/+*^ HE cells suggests that *Hhex* maintains an upstream signaling position relative to *Vegf* in subsequent hepatic cellular maturation events. This complex role of *Vegf* signaling in HE differentiation potentially involves the balanced activation of *Vegfr1/Vegfr2* transcriptional mechanisms via modulation of *Vegfa*. There is known interplay and trade-offs between VEGFR2 and VEGFR1 stimulation and it is interesting to wonder how such a dynamic could influence hepatic specification. It is clear that *Vegfa* has a direct influence on *Hhex* expression and the *Vegfa*-induced increase in *Hhex* limits the inhibitory effect that increased *Vegf* signaling has on differentiation of DE to HE in vitro. Therefore, this reciprocal relationship between *Hhex* and *Vegf* signaling (increased *Hhex* expression with reduced *Vegf*) may support a feedback mechanism that calibrates the onset and continued expression of hepatic genes resulting in a fine-tuned HE differentiation/maturation program. Future directions should aim to identify how *Hhex* and *Vegf* signaling interact to exert their independent and combined effects on the hepatic lineage. In addition, during HE differentiation the relationship between *Hhex* and *Vegfa* supports increased HE gene expression and differentiation of VEGFR2^+^ hepatic progenitors from endodermal precursors via a *Hhex*-dominant mechanism. Finally, in the absence of *Hhex*, a *Vegfa*-dominant mechanism represses HE gene expression and impairs the ability of endodermal cells to differentiate toward the hepatic lineage.

## Supporting Information

S1 Fig Differentiation Stage QPCR. *Hhex*^-/-^ HE cells did not show mRNA expression consistent with hepatic differentiation. **A)** Normalized mRNA expression of hepatic gene markers. *Hhex*^+/+^ (blue) HE cells showed dramatic increase in hepatic gene expression, while *Hhex*^-/-^ (red) cells showed a heavily attenuated expression. **B)** Normalized mRNA expression of definitive endodermal gene markers. *Hhex*^-/-^ cells fail to show a decrease in definitive endodermal gene expression characteristic of HE differentiation, as seen in *Hhex*^+/+^ cells. **C)** Normalized mRNA expression of pluripotency gene markers. *Hhex*^-/-^ cells showed increased pluripotency relative to *Hhex*^+/+^ at each differentiation stage (but particurlarly during HE differentiation) that further indicates a differentiation defect. **D)** Normalized mRNA expression of Vegf signaling gene markers. *Hhex*^-/-^ cells showed increased levels of ligands (*Vegf-a*) and receptor (*Vegfr1* and *Vegfr2*) gene expression at each differentiation stage (but particularly during HE differentiation) that indicates aberrant cellular signaling.
(TIF)

S1 Table Cell Culture. **A)** Products are listed with their manufacturer. **B)** Cell Culture recipes are listed in order of use.
(TIF)

S2 Table IF/FACS Antibodies. Antibodies are listed along with company, species, catalogue number, concentration and method used.
(TIF)

S3 Table qPCR primers. Primers are listed according to grouping of analysis (associations). Reverse primers are listed in their sense (not anti-sense) direction.
(TIF)

S4 Table Antibody Optimization. Antibodies were optimized specifically for their use in the method(s) listed. Optimal signal was chosen based on concentration and time, as well as comparison to unstained controls, Igg controls, and controls stained with secondary antibody only (background).
(TIF)
