# Supplementary figures and images for: Hhex Is Necessary for the Hepatic Differentiation of Mouse ES Cells and Acts via Vegf Signaling
Source: PLoS One. 2016 Jan 19;11(1):e0146806. doi: 10.1371/journal.pone.0146806 (PMC4718667; doi:10.1371/journal.pone.0146806)

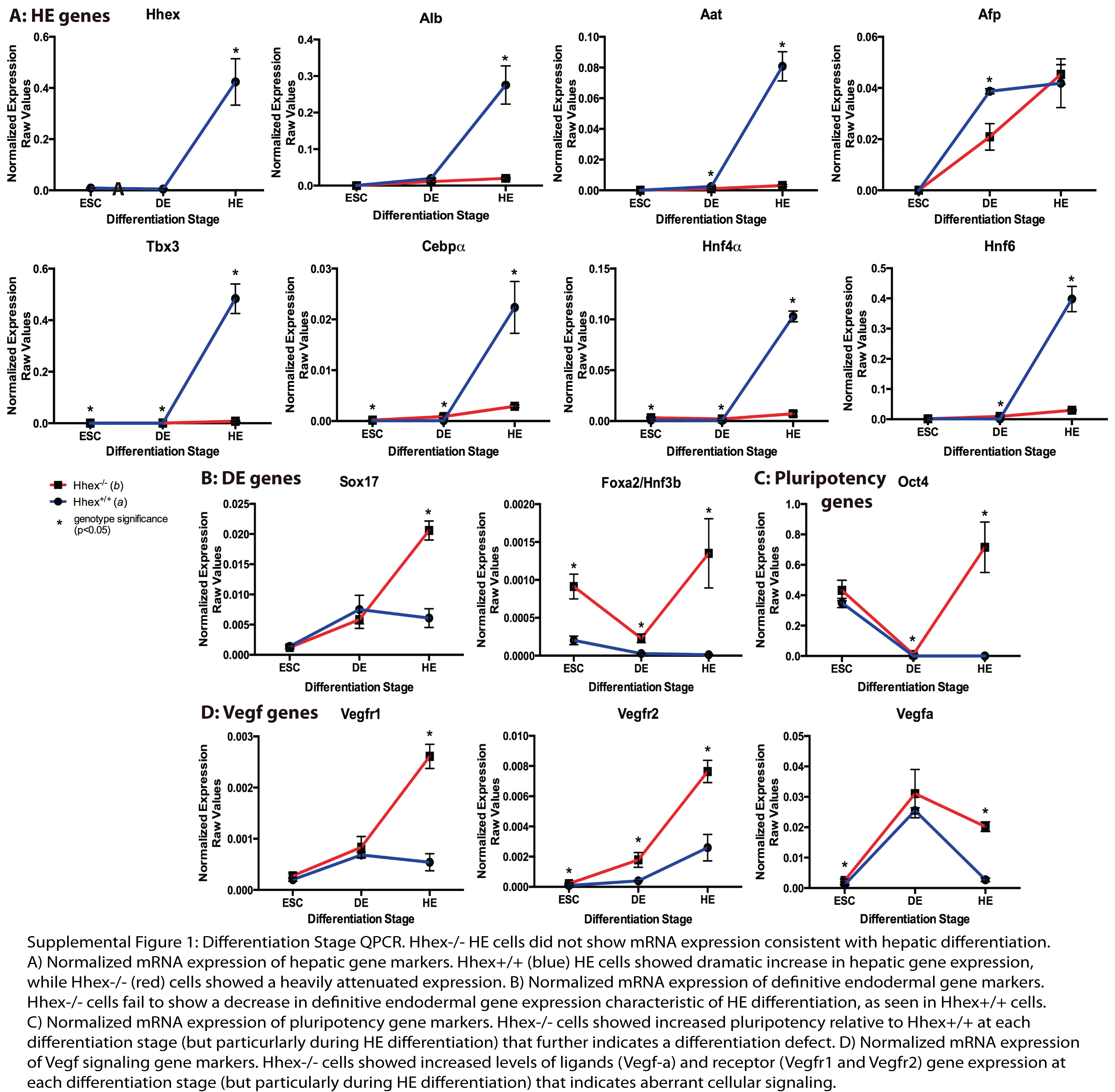

Supplement: S1 Fig — Hhex-/- HE cells did not show mRNA expression consistent with hepatic differentiation. A) Normalized mRNA expression of hepatic gene markers. Hhex+/+ (blue) HE cells showed dramatic increase in hepatic gene expression, while Hhex-/- (red) cells showed a heavily attenuated expression. B) Normalized mRNA expression of definitive endodermal gene markers. Hhex-/- cells fail to show a decrease in definitive endodermal gene expression characteristic of HE differentiation, as seen in Hhex+/+ cells. C) Normalized mRNA expression of pluripotency gene markers. Hhex-/- cells showed increased pluripotency relative to Hhex+/+ at each differentiation stage (but particurlarly during HE differentiation) that further indicates a differentiation defect. D) Normalized mRNA expression of Vegf signaling gene markers. Hhex-/- cells showed increased levels of ligands (Vegf-a) and receptor (Vegfr1 and Vegfr2) gene expression at each differentiation stage (but particularly during HE differentiation) that indicates aberrant cellular signaling. (TIF) [file pone.0146806.s001.tif]

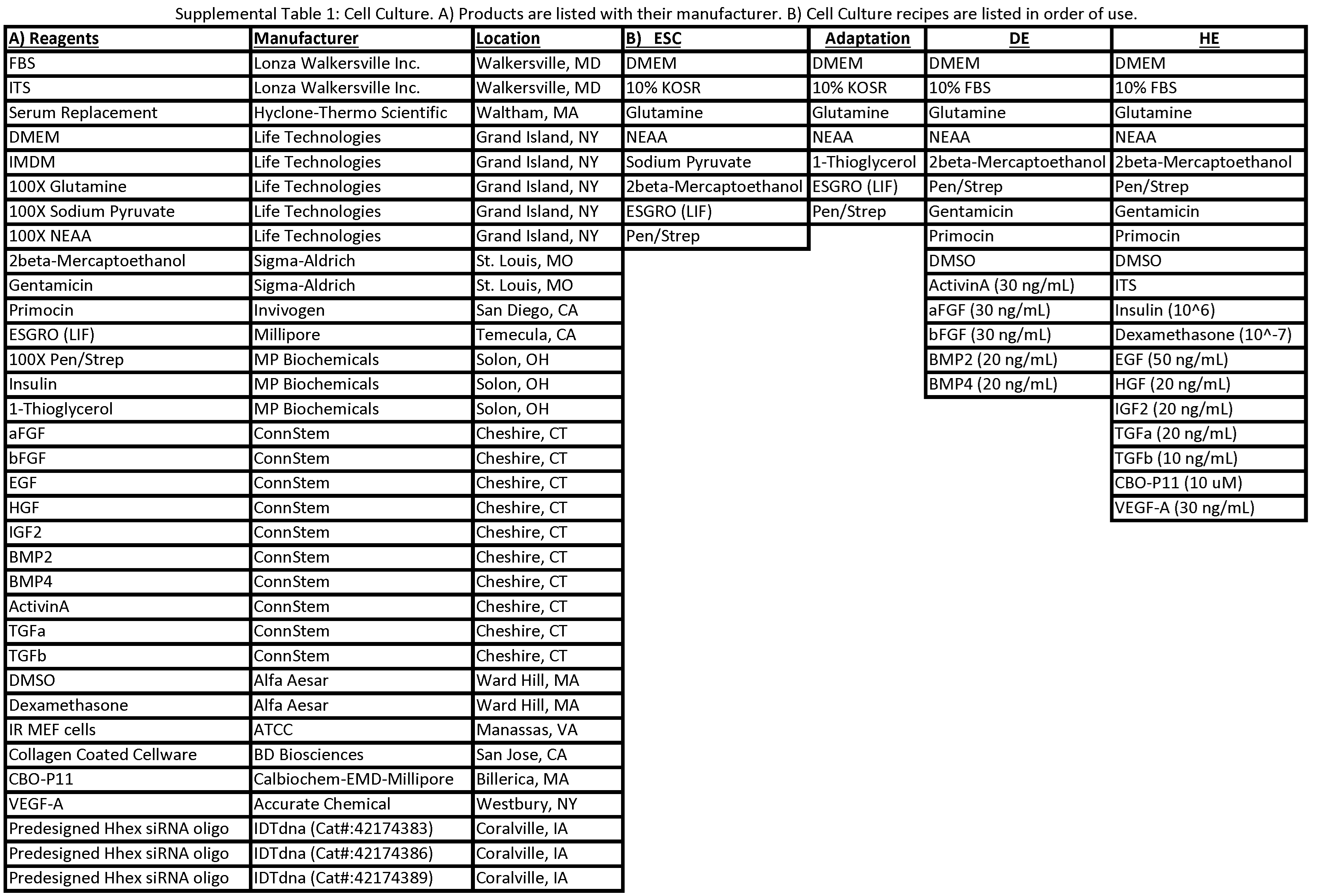

Supplement: S1 Table — A) Products are listed with their manufacturer. B) Cell Culture recipes are listed in order of use. (TIF) [file pone.0146806.s002.tif]

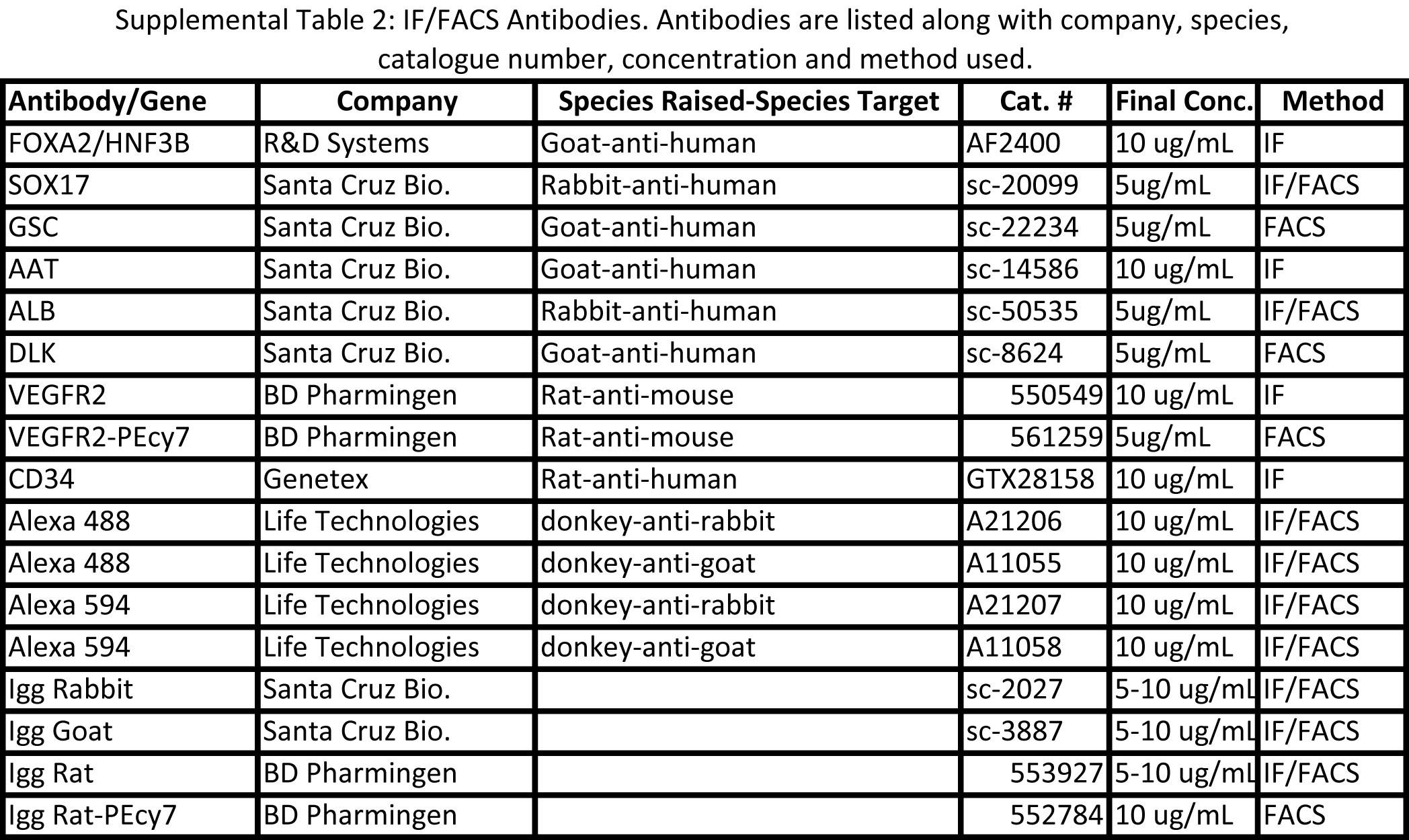

Supplement: S2 Table — Antibodies are listed along with company, species, catalogue number, concentration and method used. (TIF) [file pone.0146806.s003.tif]

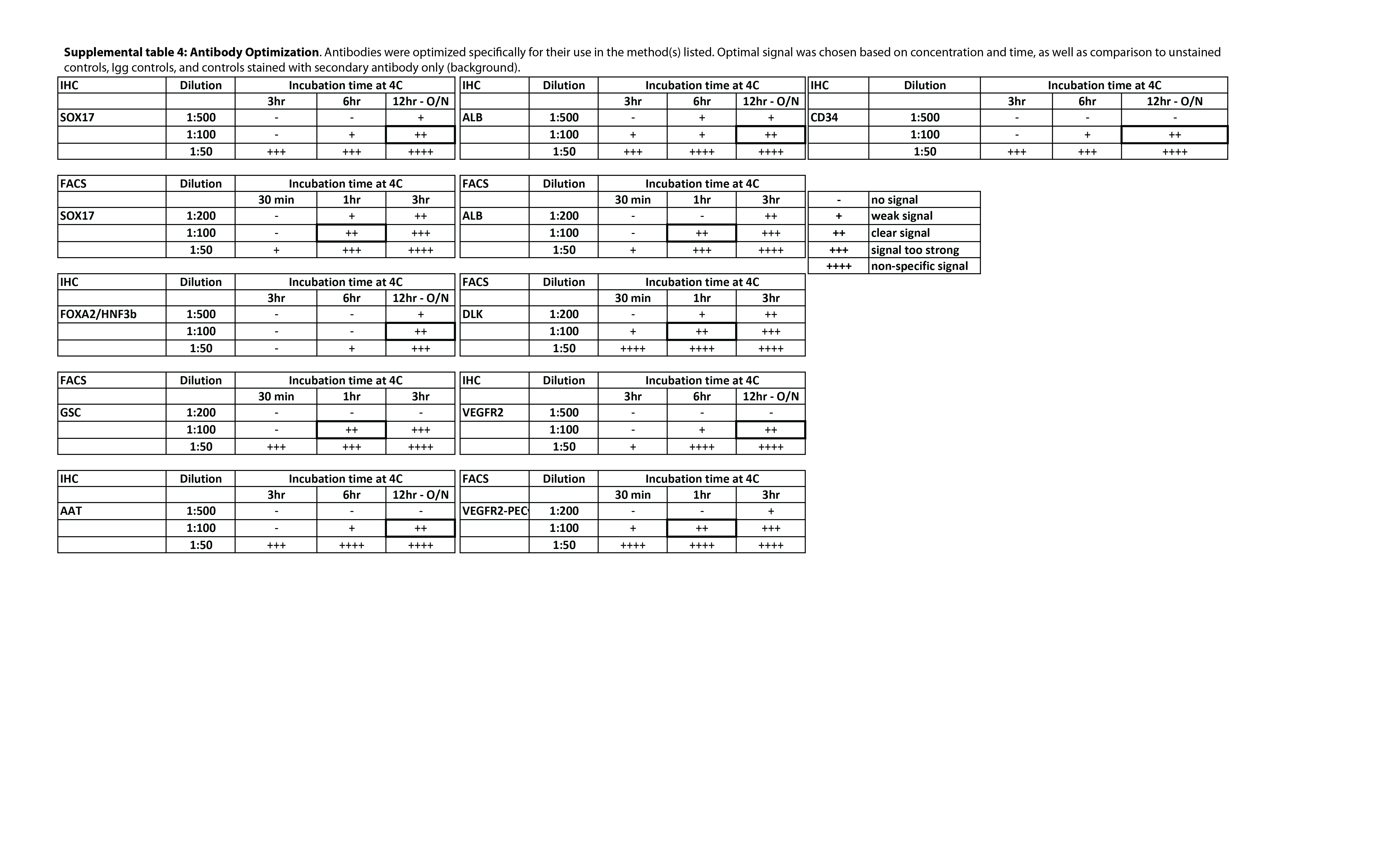

Supplement: S4 Table — Antibodies were optimized specifically for their use in the method(s) listed. Optimal signal was chosen based on concentration and time, as well as comparison to unstained controls, Igg controls, and controls stained with secondary antibody only (background). (TIF) [file pone.0146806.s005.tif]
